# Unravelling factors influencing demand for modern contraception and evaluating coverage progress since 2015 in Ethiopia, Kenya, and Nigeria: insights from multilevel and geostatistical modelling

**DOI:** 10.1186/s12889-024-19387-9

**Published:** 2024-07-11

**Authors:** McEwen Khundi, Themba Mzembe, Tabitha Ngwira, Chifuniro S Mankhwala, Chimwemwe Chifungo, Maame B Peterson, Ruth Vellemu, Nyovani J Madise, Michael G Chipeta

**Affiliations:** African Institute for Development Policy (AFIDEP), Lilongwe, Malawi

**Keywords:** Sustainable development goals, mDFPS, Women, Modern contraception, DHS, Spatial, Geostatistical

## Abstract

**Introduction:**

The United Nations established the Sustainable Development Goals (SDGs) in 2015 to enhance global development. In this study, we examine an SDG indicator: the percentage of women aged 15–49 whose family planning needs are met by modern contraception (mDFPS). We evaluate both the factors influencing its coverage and its progress since 2015.

**Methods:**

We used nationally representative surveys data (Demographic and Health Surveys (DHS) and Performance Monitoring for Action (PMA)) from Ethiopia, Kenya, and Nigeria. We assessed predictors of mDFPS. We also computed mDFPS coverage across countries and subnational areas, assessing coverage changes from the SDGs onset to the most recent period, using a Bayesian model-based geostatistical approach. We assessed whether the subnational areas exceeded the minimum recommended WHO mDFPS coverage of 75%.

**Results:**

Varied individual and community-level determinants emerged, highlighting the countries’ uniqueness. Factors such as being part of a female-headed household, and low household wealth, lowered the odds of mDFPS, while rural-residence had low odds only in Ethiopia and Nigeria. The results indicate mDFPS stagnation in most administrative areas across the three countries. Geographic disparities persisted over time, favouring affluent regions. The predicted posterior proportion of mDFPS and exceedance probability (EP) for WHO target for Ethiopia was 39.85% (95% CI: [4.51, 83.01], EP = 0.08) in 2016 and 46.28% (95% CI: [7.15, 85.99], EP = 0.13) in 2019. In Kenya, the adjusted predicted proportion for 2014 was 30.19% (95% CI: [2.59, 80.24], EP = 0.06) and 44.16% (95%CI: [9.35, 80.24], EP = 0.13) in 2022. In Nigeria, the predicted posterior proportion of mDFPS was 17.91% (95% CI: [1.24, 61.29], EP = 0.00) in 2013, and it was 23.08% (95% CI: [1.80, 56.24], EP = 0.00) in 2018. None of the sub-national areas in Ethiopia and Nigeria exceeded the WHO target. While 9 out of 47 counties in Kenya in 2022 exceeded the WHO mDFPS target.

**Conclusion:**

The study unveils demographic, geographic, and socioeconomic mDFPS disparities, signalling progress and stagnation across administrative areas. The findings offer policymakers and governments insights into targeting interventions for enhanced mDFPS coverage. Context-specific strategies can address local needs, aiding SDG attainment.

**Supplementary Information:**

The online version contains supplementary material available at 10.1186/s12889-024-19387-9.

## Introduction

The United Nations member states adopted the Agenda 2030 for Sustainable Development Goals (SDGs) in 2015. The agenda has 17 goals building on the Millennium Development Goals (MDGs) to stimulate action toward shared sustainable prosperity. The use of modern contraceptive methods falls under SDG 3.7, which states that “*by 2030*,* countries should ensure universal access to sexual and reproductive health (SRH) care services for family planning*,* information*,* and education*,* and the integration of reproductive health into the national strategies and programmes*” [47]. Several indicators track this goal, one of which is indicator 3.7.1, which measures the percentage of women of reproductive age (15–49 years) who have their demand for family planning satisfied with modern contraceptive methods (mDFPS) [47].

Globally, an estimated 1.1 billion women of reproductive age needed family planning to postpone or avoid getting pregnant in 2020 [48]. However, only 851 million had access to and used modern contraceptives, and an additional 85 million used traditional contraception methods. Modern contraception methods are defined as any product or medical procedure that prevents pregnancy from occurring as a result of sexual intercourse [25]. Among those women who were using contraception in 2020, 90% of them were using a modern method [48]. Modern contraceptive methods are considered more effective at preventing pregnancy than traditional methods, and many health facilities encourage their use (Sully et al. 2020). Sub-Saharan Africa (SSA) has the lowest proportion of modern contraceptive use among all women of childbearing age, with just 22.0% (95% Confidence interval: [21.8, 22.2]) [11]. In 2020, among women who wanted to postpone pregnancy, only 55% were using modern contraceptives in SSA. In addition, most countries with lower than 50% mDFPS were from the region [14, 48].

Women who can make informed decisions about their sexual relations, contraceptive use and reproductive health are more likely to use modern contraceptives to meet their family planning needs [6]. In turn, meeting the demand for family planning with modern contraception methods empowers women to prevent unintended and high-risk pregnancies, thereby lowering the risk of maternal and under-five mortality [16, 45]. Additionally, modern contraception reduces the risk of early childbearing and allows women to achieve their educational goals. Thus, access to and use of modern contraception can improve women’s health and educational opportunities, hence improving women’s chances of paid labour employment [9, 40].

Since the inception of the SDGs in 2015, it has been imperative that countries track their progress within different demographic, social, economic, and geographical groups to ensure that no one is left behind [5, 47]. Countries can identify disparities in access to modern contraceptives and other services by monitoring this progress. They can then use this information to address these disparities and ensure that all women of reproductive age can access the necessary information and services to achieve their desired family planning needs with modern contraception methods [47].

The current analysis focuses on Ethiopia, Kenya, and Nigeria, with Nigeria and Ethiopia being the most populous nations in Africa and Kenya ranking seventh [51]. Kenya ranks among the top countries in SSA regarding the modern contraceptive use prevalence rate (mCPR) among married women, at 56.9%. Ethiopia, on the other hand, has a moderate mCPR rate of 40.5%, whereas Nigeria has one of the lowest prevalence rates among married women in SSA, at 12% [11, 19, 37]. In terms of the proportion of mDFPS among married women, coverage was 64.9% in Ethiopia, 74.7% in Kenya, and 33.9% in Nigeria [19, 37, 48].

In addition, there is an unequal distribution of modern contraceptive use among various categories within these countries. Adolescents and young women (ages 15 to 24), economically disadvantaged women, women with limited education (below secondary school level), and rural residents have lower utilisation rate [2, 10, 12]. Although the predictors of modern contraceptive utilisation have been investigated, there needs to be more literature regarding the predictors of mDFPS [5]. Hypothesising that similar factors influence the adoption of modern contraception and mDFPS, this study seeks to investigate these factors and evaluate and compare the progress in mDFPS coverage since 2015 in the three countries.

## Methods

### Data sources

The study used data from Demographic and Health Surveys (DHS) and Performance Monitoring for Action (PMA) from three countries, namely Ethiopia, Nigeria and Kenya [19, 30, 31, 36, 54]. The DHS is a nationally representative, cross-sectional survey that provides data on various health indicators, including family planning. PMA surveys cover a subset of the variables included in the DHS and are done in selected administrative areas in each country. DHS data, collected closer to the inception of the SDGs in 2015, was utilised. For more recent data where DHS was unavailable, the PMA survey data was utilised. Data from Ethiopia was obtained from the 2016 DHS survey and the 2019 PMA survey, excluding the 2019 mini-DHS survey due to the absence of necessary questions for calculating mDFPS [18, 54]. Kenya data was analysed using the DHS for 2014 and 2022 [30, 31]. Nigeria’s data analysis relied on the 2013 and 2018 DHS surveys [36, 37].

The outcome of interest was defined as a binary variable (Yes/No): where women 15–49 years old who needed family planning and were using modern contraception methods to satisfy that need were considered to have demand for family planning satisfied with modern contraception methods (Yes); the need for family planning was defined as fecund women who either needed to space or limit childbearing [5, 20]. By choosing to use mDFPS instead of just modern contraception prevalence, we were able to restrict the analysis to individuals with a demand for contraception, making it easier to track the progress of SDG indicator 3.7.1 [5, 20]. Modern contraceptive methods include pills, condoms (male and female), injectables, hormonal implants, patches, diaphragms, spermicidal agents (foam/gel) and emergency contraception.

### Statistical analysis

We adjusted exploratory statistics for complex survey design [34].

### Multilevel modelling

Using the latest standard DHS survey datasets for each country, we used a multilevel Bayesian logistic model to fit the data to explore factors associated with the outcome of interest. Multilevel models were used because of the hierarchical nature of the data’s sampling framework (Eq. 1) [44]. All Women from sampled households in enumeration areas (clusters) are selected for inclusion. The enumeration areas are, in turn, located in administrative areas (counties, regions or states). The independent variables included in the adjusted models were selected based on the literature, to allow within and between country comparison [20, 44]. The following variables were included in the multilevel model: place of residence (rural versus urban), age category, respondent’s level of education, household head gender, wealth quintile, marital status and religion and parity.

1 $$\begin{aligned}\mathrm{logit}(P\left({x}_{kji}\right))&={\beta }_{0}+{\beta }_{1}{{\varvec{X}}}_{k:\left(j:i\right)}\dots +{region}_{i}+{cluster}_{j:i}\\\text{i}&=1,\dots ,{n}_{c};\text{j}=1...,{n}_{h};\text{k}=1,..,{n}_{w}\end{aligned}$$

Where the indices are defined for region $\:i$, cluster *j* and individual $\:k$. Index *ji* denotes that cluster *j* is nested within a region $\:i.$ Similarly, index *k: (j: i)* denotes that individual *k* is nested within a cluster (whereupon the cluster is also nested within a region $\:i$). The vector ***X*** denotes the covariates, and $\:\beta\:s\:$are the coefficients for the covariates.

### Geostatistical modelling

A model-based Bayesian geostatistical logistic model to derive and assess the geographical variation in coverage for mDFPS (Eq. 2) [32] was also used in the study. The variables used to adjust for confounding in these models were informed by the variables identified in the multilevel modelling stage and the literature. We included the variables in the analysis based on the availability of the spatial raster data of those variables for the countries of interest. The raster data included in the geostatistical models were women’s education years, population density, probability of seeking care at health facilities, poverty rate and mean parity [52].

2 $$\:logit\left(P\left({x}_{i}\right)\right)={\beta\:}_{0}+{\beta\:}_{1}{\varvec{X}}_{i}+\dots\:+{S(x}_{i})$$

Where the vector $\:\varvec{X}$ denotes covariates and $\:\beta\:s$ are coefficients for the covariates conditional on the true prevalence $\:\varvec{P}\left({\varvec{x}}_{\varvec{i}}\right)$ at location $\:{\varvec{S}(\varvec{x}}_{\varvec{i}}$), $\:\varvec{i}$ = 1, …, n, where the number of positive results is $\:{\varvec{x}}_{\varvec{i}}\:$ out of $\:{\varvec{N}}_{\varvec{i}}\:$ with a binomial distribution. $\:\varvec{S}(.)$ is a spatial random effect that follows a zero-mean Gaussian process with the Matérn covariance function.

The geostatistical models were fit to two time points, at the start, closest to 2015 and end, the most recent, to allow comparison in the coverage of mDFPS between the two-time points to assess progress. Posterior samples were drawn based on the models to calculate sub-national-level mDFPS estimates and 95% credible intervals (CI) for each sub-national area. In addition, the predicted posterior samples were used to estimate the mean posterior change (MPC) of the outcome of interest between the years on which the geostatistical models were based to assess the change (progress) in coverage of mDFPS. Given the available data, we quantified how likely it was for the coverage of mDFPS to be above the recommended WHO minimum coverage of 75% [47] for each sub-national area (region, state or county), set as threshold ***t***. This was achieved by quantification of the exceedance probability (EP) presented in Eq. 3. An EP close to 1 indicates that the prevalence of the outcome was above ***t***, EP close to 0 indicates that the prevalence was likely below ***t***. An EP value of around 0.5 indicated that the prevalence was equally likely to be below or above ***t***, hence the high uncertainty [32]. Equation 3 was also used to calculate EPs for MPC between the two time periods to assist in assessing if there was an improvement in coverage between the two times. In this case, the threshold ***t*** in Eq. 3 was set to zero [8].

3 $$\:EP=Probability\left(P\left({\varvec{x}}_{\varvec{i}}>\varvec{t}\:|\:data\right)\right)$$

For each country, we generated coverage of the outcome, and EP maps at 5 by 5 km pixel-, sub-national-, and national levels to influence decision-making at various administrative levels. These maps show areas where coverage is the lowest or highest. These maps and tables would enable program implementers to appreciate where progress is lagging and to prioritise health interventions in areas that need them most while maintaining support where coverage is already good [29]. R programming software version 4.2.1 was utilised for data management, and the Bayesian models were fitted using the INLA R package [32].

## Results

The Ethiopian DHS 2016 survey had 5,312 women aged 15–49 who expressed a desire for family planning. The sample was drawn from 606 clusters. Similarly, the Ethiopian PMA 2019 survey included 3,396 women, sampled from 265 clusters. The 2014 Kenyan DHS had 7,840 women with a demand for family planning, selected from 1,549 clusters. The 2022 Kenyan DHS survey comprised 8,911 women with a demand for family planning, drawn from 1,662 clusters. The 2013 Nigerian DHS encompassed 11,464 women with data on the demand for family planning, sampled from 887 clusters, while the 2018 survey included 12,243 women with a demand for family planning, drawn from 1,376 clusters.

### Respondent characteristics and mDFPS coverage

According to the latest standard DHS surveys of Ethiopia 2016, Kenya 2022 and Nigeria 2018, the majority of respondents were rural residents in Ethiopia (80.99%) and Kenya (59.75%) while in Nigeria rural residents were (46.27%). About 20% of respondents were aged 15–24, Ethiopia (23.31%), Kenya (24.12), and Nigeria (20.42%). Refer to Table 1 for more details on the characteristics of the respondents.

**Table 1:** Characteristics of respondents based on the latest DHS data. Both counts and percentages were weighted to adjust for the survey design

Characteristic	Ethiopia (year: 2016)	Kenya (year:2022)	Nigeria (year: 2018)
	*N* = 6,356	*N* = 9,490	*N* = 12,331
**Residence**			
Urban	1,208 (19.01%)	3,819 (40.25%)	6,625 (53.73%)
Rural	5,148 (80.99%)	5,671 (59.75%)	5,705 (46.27%)
**Age groups**			
15–24	1,482 (23.31%)	2,289 (24.12%)	2,517 (20.42%)
25–34	2,917 (45.89%)	3,777 (39.80%)	4,880 (39.58%)
35–49	1,957 (30.80%)	3,424 (36.08%)	4,933 (40.01%)
**Level of education**			
Secondary or higher	799 (12.56%)	5,261 (55.44%)	7,339 (59.52%)
Primary	1,931 (30.38%)	3,859 (40.66%)	2,053 (16.65%)
No education	3,626 (57.05%)	370 (3.90%)	2,939 (23.83%)
**Sex of household head**			
Male	5,357 (84.29%)	6,296 (66.34%)	10,347 (83.91%)
Female	999 (15.71%)	3,194 (33.66%)	1,984 (16.09%)
**Wealth index**			
Poorest	970 (15.26%)	1,344 (14.17%)	1,339 (10.86%)
Poorer	1,263 (19.87%)	1,725 (18.17%)	1,771 (14.36%)
Middle	1,326 (20.87%)	1,797 (18.93%)	2,395 (19.42%)
Richer	1,294 (20.36%)	2,188 (23.06%)	3,253 (26.38%)
Richest	1,503 (23.65%)	2,436 (25.67%)	3,573 (28.98%)
**Marital status**			
In union	5,949 (93.60%)	7,089 (74.70%)	10,337 (83.83%)
Never in union	138 (2.18%)	1,394 (14.69%)	1,624 (13.17%)
Formerly in union	269 (4.23%)	1,007 (10.62%)	369 (2.99%)
**Religion group**			
Islam	1,883 (29.63%)	363 (3.82%)	5,077 (41.17%)
Orthodox	2,896 (45.57%)		
Roman Catholic		1,863 (19.64%)	1,458 (11.83%)
Other	79 (1.24%)	372 (3.92%)	45 (0.37%)
Other christians	1,497 (23.56%)	6,891 (72.62%)	5,750 (46.63%)
**Parity group**			
None	552 (8.68%)	948 (9.99%)	1,440 (11.68%)
1–2	1,807 (28.42%)	3,837 (40.43%)	2,991 (24.26%)
3–4	1,598 (25.14%)	2,999 (31.60%)	3,301 (26.77%)
5+	2,399 (37.75%)	1,706 (17.98%)	4,598 (37.29%)

The 95% crude confidence interval (CCI) mDFPS coverage was 61.35% 95% CCI: (58.38, 64.23) (in 2016 DHS) and 62.41% 95% CCI: (58.76, 65.92) (in 2019 PMA) in Ethiopia, 70.81% 95% CCI: (69.38, 72.21) (in 2014 DHS) and 74.59% 95% CCI: (73.35, 75.79) (in 2022 DHS) in Kenya, while in Nigeria, it was 38.82% 95% CCI: (36.74, 40.95) (in 2013) and 35.66% 95% CCI: (34.40, 36.93) (in 2018). Urban residents had higher coverage of mDFPS coverage in all countries: Ethiopia (78.17%), Kenya (75.66%) and Nigeria (40.46%). Refer to Table 2 for more details on mDFPS coverage. The unadjusted weighted sub-national level estimates of the mDFPS coverage are in supplemental Figures S1 to S3.

**Table 2:** Coverage of demand for family planning satisfied with modern methods (mDFPS) by characteristics of respondents based on latest DHS data. Both counts and percentages were weighted to adjust for survey design. Additional details are in tables S4 to S6

	Ethiopia (year: 2016)	Kenya (year:2022)	Nigeria (year: 2018)
**Residence**			
Urban	78.17%	75.66%	40.46%
Rural	57.40%	73.87%	30.08%
**Age groups**			
15–24	65.51%	67.99%	32.57%
25–34	63.18%	78.04%	37.47%
35–49	55.47%	75.19%	35.44%
**Level of education**			
Secondary or higher	76.64%	74.79%	42.00%
Primary	64.13%	77.09%	35.54%
No education	56.50%	45.64%	19.90%
**Sex of household head**			
Male	61.69%	75.98%	35.25%
Female	59.53%	71.85%	37.76%
**Wealth index**			
Poorest	43.41%	65.55%	18.70%
Poorer	53.37%	76.64%	26.77%
Middle	60.67%	76.22%	33.53%
Richer	67.32%	74.87%	39.72%
Richest	75.09%	76.66%	44.14%
**Marital status**			
In union	60.58%	74.72%	33.88%
Never in union	63.14%	69.33%	43.51%
Formerly in union	77.46%	80.96%	50.77%
**Religion group**			
Islam	42.64%	54.96%	28.62%
Orthodox	70.73%		
Roman Catholic		73.97%	37.83%
Other	27.07%	67.06%	15.97%
Other christians	68.54%	76.19%	41.47%
**Parity group**			
None	68.47%	56.14%	42.23%
1–2	72.47%	78.28%	36.03%
3–4	66.03%	78.57%	39.45%
5+	48.23%	69.54%	30.63%

### Multilevel modelling

The results of the multilevel Bayesian logistic model varied between countries. This analysis was based on the latest standard DHS from each of the three countries for easier comparison between countries. In Ethiopia and Nigeria, women residing in rural areas had significantly lower odds of mDFPS compared to women residing in urban areas. In Ethiopia, the OR was 0.59 (95% CI: [0.42, 0.82]), implying 41% lower odds of mDFPS among rural women. In Nigeria, the OR was 0.88 (95% CI: [0.78, 0.99]), signifying 12% decreased odds of mDFPS among rural women. But in Kenya, the OR of mDFPS was not significantly different between rural and urban residents; (OR: 0.91, 95% CI: [0.78, 1.06]). Detailed information is presented in Table 3.

**Table 3:** Table of adjusted odds ratios (aOR) for demand for family planning satisfied by modern contraception methods (mDFPS) in Ethiopia, Kenya and Nigeria using bayesian logistic regression (eq. 1) based on latest DHS data

	Ethiopia (year: 2016)	Kenya (year: 2022)	Nigeria (year: 2018)
	aOR (95% CI)	aOR (95% CI)	aOR (95% CI)
**Residence (urban)**			
Rural	0.59 (0.42, 0.82)	0.91 (0.78, 1.06)	0.88 (0.78, 0.99)
**Age groups (35–49 years)**			
15–24 years	1.06 (0.81, 1.37)	0.92 (0.77, 1.1)	0.75 (0.64, 0.88)
25–34 years	1.18 (0.98, 1.40)	1.2 (1.05, 1.36)	0.98 (0.88, 1.08)
**Level of education (Secondary or higher)**			
No education	1.09 (0.85, 1.38)	0.40 (0.31, 0.50)	0.51 (0.44, 0.59)
Primary	1.14 (0.92, 1.41)	1.06 (0.94, 1.21)	0.89 (0.79, 1.00)
**Household head sex (Male)**			
Female	0.62 (0.52, 0.74)	0.73 (0.65, 0.82)	0.82 (0.73, 0.93)
**Wealth index (Richest)**			
Poorest	0.23 (0.17, 0.32)	0.73 (0.58, 0.92)	0.49 (0.4, 0.61)
Poorer	0.4 (0.29, 0.54)	1.11 (0.89, 1.38)	0.56 (0.47, 0.66)
Middle	0.55 (0.4, 0.74)	1.12 (0.92, 1.37)	0.77 (0.67, 0.89)
Richer	0.74 (0.55, 0.99)	1.05 (0.89, 1.24)	0.94 (0.84, 1.06)
**Marital status (In union)**			
Never in union	0.80 (0.53, 1.20)	1.41 (1.16, 1.72)	1.51 (1.21, 1.87)
Formerly in union	3.18 (2.15, 4.78)	2.00 (1.64, 2.44)	2.19 (1.74, 2.77)
**Religion group (Islam)**			
Other	0.64 (0.3, 1.33)	1.81 (1.32, 2.48)	0.97 (0.47, 1.89)
Orthodox	1.91 (1.55, 2.35)		
Roman Catholic		2.07 (1.6, 2.67)	1.35 (1.13, 1.62)
Other Christians	1.77 (1.38, 2.28)	2.19 (1.72, 2.78)	1.45 (1.28, 1.65)
**Parity groups (3–4)**			
None	1.09 (0.80, 1.50)	0.38 (0.29, 0.49)	0.92 (0.72, 1.17)
1–2	1.49 (1.22, 1.82)	0.89 (0.77, 1.03)	0.86 (0.76, 0.97)
5+	0.64 (0.53, 0.77)	0.78 (0.67, 0.9)	0.98 (0.88, 1.10)
**Cluster-level variance**	0.47 (0.35, 0.62)	0.10 (0.04, 0.17)	0.20 (0.15, 0.27 )
**Regional/county/state-level variance**	0.80 (0.28, 1.92)	0.15 (0.07, 0.27)	0.32 (0.19, 0.53)

In Ethiopia, women with secondary school or higher level of education did not have significantly different odds of mDFPS when compared to women with primary school education and no education (OR: 1.09, 95% CI: [0.85, 1.38]) and (OR: 1.14, 95% CI: [0.92, 1.41]). However, in Kenya, only women with no education had lower odds of mDFPS (OR: 0.40, 95% CI: [0.31, 0.50]) versus women with a secondary school or higher-level education. Similarly, in Nigeria, only women who did not have any education had lower odds of mDFPS (OR: 0.51, 95% CI: [0.44, 0.59]) versus women with a secondary of higher-level education (Table 3). These findings suggest that the relationship between education level and mDFPS varies across the three countries, with education being positively associated with higher mDFPS in Kenya and Nigeria but not in Ethiopia.

In terms of marital status, women who had never been in a union had higher odds of mDFPS compared to those who were in a union in Kenya (OR: 1.41, 95% CI: [1.16, 1.72]) and Nigeria (OR: 1.51, 95% CI: [1.21, 1.87]), but not in Ethiopia (odds ratio: 0.80, 95% CI: 0.53, 1.20). Furthermore, women who were no longer in a relationship had higher odds of mDFPS compared to those who were in a union: Ethiopia (OR: 3.18, 95% CI: [2.15, 4.78]), Kenya (OR: 2.00, 95% CI: [1.64, 2.44]), and Nigeria (OR: 2.19, 95% CI: [1.74, 2.77]) (Table 3).

In comparison with Muslim women, those who identified themselves as Orthodox, Roman Catholic or Protestants had higher odds of mDFPS. Women with a parity of 3–4 did not have significantly different odds of mDFPS to those with a parity of none in Ethiopia and Nigeria, while in Kenya women with a parity of none had lower odds of mDFPS (OR: 0.38, 95% CI: [0.29, 0.49]). Women with a parity of 1–2 had higher odds of mDFPS in Ethiopia (OR: 1.49, 95% CI: [1.22, 1.82]), while women with a parity of five or more had lower odds of mDFPS in Ethiopia (OR: 0.64, 95% CI: [0.53, 0.77]) and Kenya (OR: 0.78, 95% CI: [0.67, 0.90]). Furthermore, in all three countries, there were substantial residual variation at both the cluster level and the regional, county, or state level, after accounting for the confounding variables (Table 3).

### Geostatistical modelling

The results of the Bayesian geostatistical logistic regression based on the latest survey are presented in Table 4. The implemented models were adjusted to account for spatial confounding by their definition (Eq. 2). Notably, in these models, an increase in women’s education years was consistently associated with higher odds of mDFPS in all three countries. The highest odds were observed in Nigeria (odds ratio: 8.49, 95% CI: [5.03, 14.35]), followed by Ethiopia (OR: 2.69, 95% CI: [1.39, 5.11]), and Kenya (OR: 2.63, 95% CI: [1.79, 3.65]). The results of the Bayesian geostatistical model for the first survey are in the Supplementary Table S3.

**Table 4:** Adjusted odds ratios (aOR) for demand for family planning satisfied with modern contraception methods in Ethiopia (PMA), Kenya (DHS) and Nigeria (DHS) based on the latest health surveillance survey using bayesian geostatistical logistic regression (eq. 2)

Characteristic	Ethiopia (year: 2019)	Kenya (year: 2022)	Nigeria (year: 2018)
	aOR (95% CI)	aOR (95% CI)	aOR (95% CI)
**Women education years**	2.69 (1.39, 5.11)	2.63 (1.79, 3.65)	8.49 (5.03,14.35)
**Population density**	1.00 (0.89, 1.13)	1. 06 (0.97, 1.08)	1.06 (1.01, 1.12)
**Mean parity**	0.72 (0.66, 0.79)	0.94 (0.90, 0.99)	0.93 (0.90, 0.96)
**Probability seeking care at a health facility**	1.77 (0.75, 4.23)	0.52 (0.25, 1.05)	1.20 (0.92, 1.56)
**Percentage of people living below a one USA dollar** ^**1**^		0.32 (0.18, 0.57)	1.17 (0.48, 2.83)

^1^Percentage of people living below US $1-dollar variable was not available for Ethiopia, *PMA *Performance Monitoring for Action, *DHS *Demographic Health Survey

**Fig. 1:**
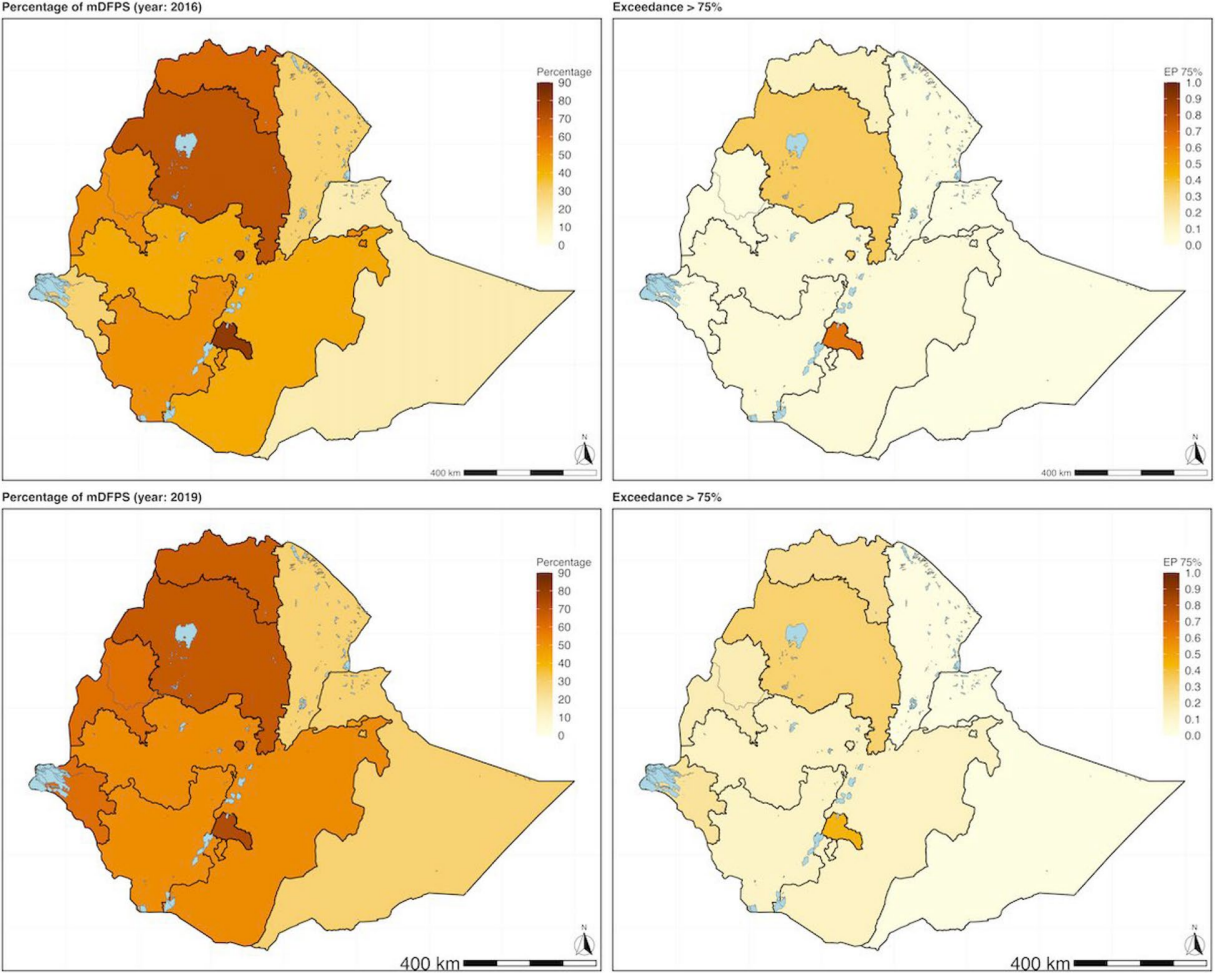
Proportion of demand for family planning satisfied with modern contraception methods (mDFPS) for Ethiopia. Estimates based on adjusted Bayesian geostatistical models. ***Top left panel***: Map of the predicted proportion of demand for family planning satisfied with modern contraception methods (mDFPS) in Ethiopia in 2016 (DHS). ***Top right panel***: The likelihood or certainty that the estimated mDFPS exceeds the 75% threshold (SDG target of mDFPS). ***Bottom left panel***: Map of the predicted proportion of mDFPS in Ethiopia in 2019 (PMA). ***Bottom right panel***: The likelihood or certainty that the estimated mDFPS exceeds the 75% threshold (SDG target of mDFPS)

**Fig. 2:**
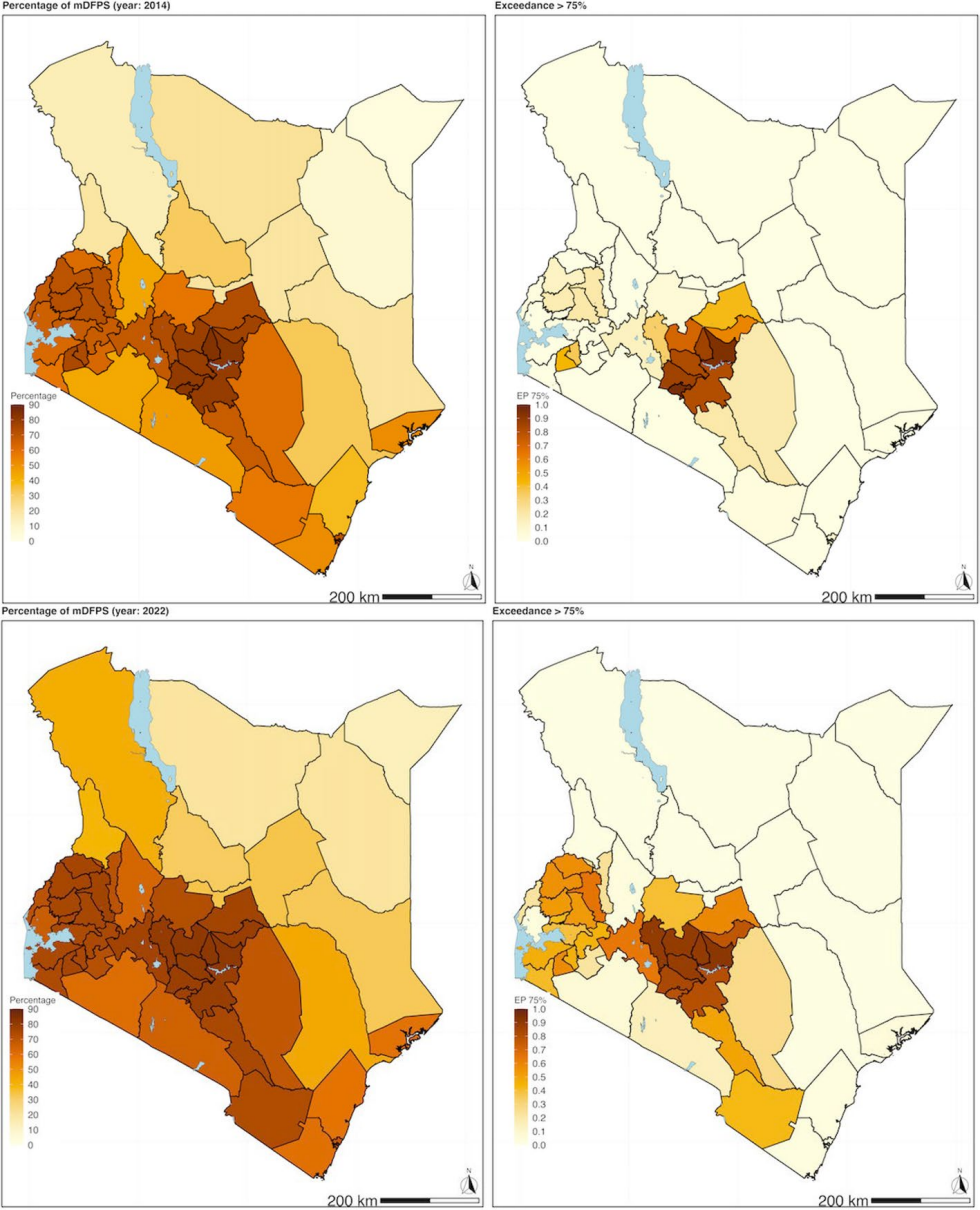
Proportion of demand for family planning satisfied with modern contraception methods (mDFPS) for Kenya. Estimates based on adjusted Bayesian geostatistical models. ***Top left panel***: Map of the predicted proportion of demand for family planning satisfied with modern contraception methods (mDFPS) in Kenya in 2014 (DSHS). ***Top right panel***: The likelihood or certainty that the estimated mDFPS exceeds the 75% threshold (SDG target of mDFPS)​. ***Bottom left panel***: Map of the predicted proportion of mDFPS in Kenya in 2022 (DHS).​ ***Bottom right panel***: The likelihood or certainty that the estimated mDFPS exceeds the 75% threshold (SDG target of mDFPS).​

**Fig. 3:**
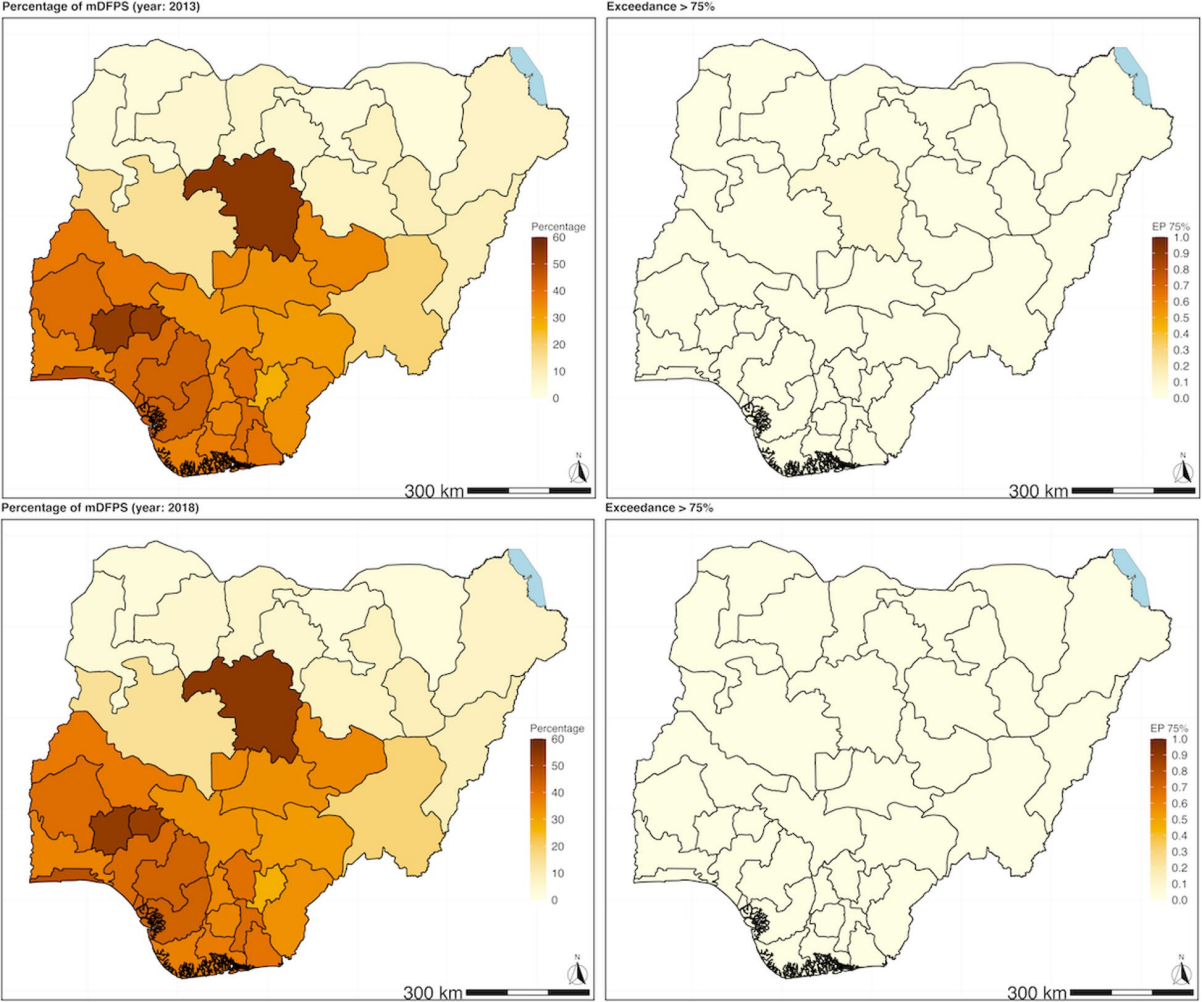
Proportion of demand for family planning satisfied with modern contraception methods (mDFPS) for Nigeria. Estimates based on adjusted Bayesian geostatistical models. ***Top left panel***: Map of the predicted proportion of demand for family planning satisfied with modern contraception methods (mDFPS) in Ethiopia in 2013 (DHS). ***Top right panel***: The likelihood or certainty that the estimated mDFPS exceeds the 75% threshold (SDG target of mDFPS)​. ​***Bottom left panel***: Map of the predicted proportion of mDFPS in Ethiopia in 2018 (DHS). ***Bottom right panel***: The likelihood or certainty that the estimated mDFPS exceeds the 75% threshold (SDG target of mDFPS).​

The predicted posterior samples of the proportion of mDFPS from the geostatistical models were used to map the adjusted mDFPS of the three countries between the two time periods (Figs. 1, 2 and 3). The adjusted predicted posterior proportion of mDFPS and EP for exceeding the 75% WHO target for Ethiopia was 39.85% (95% CI: [4.51, 83.01], EP = 0.09) in 2016 and 46.28% (95% CI: [7.18, 85.99], EP = 0.13) in 2019. In Kenya, the adjusted predicted proportion for 2014 was 30.19% (95% CI: [2.59, 80.24], EP = 0.06) and 44.16% (95%CI: [9.35, 80.24], EP = 0.13) in 2022. In Nigeria, the predicted posterior proportion of mDFPS was 17.90% (95% CI: [1.24, 61.28], EP = 0.00) in 2013, and it was 23.08% (95% CI: [1.80, 56.24], EP = 0.00) in 2018.

The pattern of mDFPS coverage was similar between the two time periods for all three countries, with some exceptions (Figs. 1, 2 and 3). None of regions in Ethiopia had an EP of greater than or equal to 0.90, surpassing 75% WHO recommended coverage in either period. In Kenya, in 2014, seven counties (Embu, Kiambu, Kirinyaga, Machakos, Murang’a, Nairobi and Nyeri) had an EP of greater than or equal to 0.9 that they exceeded the 75% WHO recommended coverage, while in 2022 there were only seven (Embu, Kiambu, Kirinyaga, Machakos, Murang’a, Nyandarua, Nyeri), with an additional two counties (Nairobi and Tharaka-Nithi) that had an EP of above 0.8. In Nigeria, none of the states had an EP probability greater than or equal to 0.9 in either survey year in 2013 and 2018.

In addition, the geostatistical predictive posterior samples from the geostatistical models were used to calculate the mean posterior change (MPC) in the proportion of mDFPS between the two time periods for each included country. The MPC was calculated overall for the country and at the sub-national level (Table 5, Supplementary material Tables S1 & S2). An EP was also calculated, interpreted as the probability that the MPC between the first and the second period was greater than zero; in other words, to assess whether mDFPS coverage improved in the second period. The overall national level 95% CI of the absolute change in the proportion of mDFPS for the three countries were as follows: Ethiopia: 5.68% (95% CI: [-38.07, 49.44], EP = 0.61); Kenya: 10.19% (95% CI: [-17.72, 39.33], EP = 0.80) and Nigeria: 1.98% (95% CI: [-26.48, 34.49], EP = 0.58).

**Table 5:** Table showing Ethiopia’s regional proportion of mDFPS (2016–2019) with 95% credible intervals (CI), mean posterior change (MPC), percentage change, and exceedance probability of increase of greater than zero of mDFPS from 2016 to 2019. Estimates based on adjusted bayesian geostatistical models

Region	% mDFPS 2019 (95% CI)	% mDFPS 2016 (95% CI)	Mean posterior change (MPC) in mDFPS (95% CI)	Probability of increase of mDFPS from 2016 to 2019
Addis Ababa	69.92 (56.53, 78.94)	72.82 (61.41, 80.81)	-2.81 (-16.88, 9.86)	0.32
Afar	28.48 (6.16, 71.17)	29.16 (6.68, 75.2)	-1.24 (-44.57, 45.33)	0.48
Amhara	68.2 (29.1, 89.26)	69.9 (34.55, 88.23)	-1.74 (-38.43, 33.46)	0.46
Benishangul Gumz	60.45 (22.76, 86.47)	50.97 (15.47, 75.18)	9.52 (-31.92, 52.58)	0.68
Dire Dawa	46.33 (15.57, 66.99)	51.2 (26.92, 65.48)	-4.72 (-30.01, 16.9)	0.33
Gambela	60.79 (23.48, 88.94)	28.16 (8.32, 73.22)	28.26 (-14.73, 66.86)	0.90
Harari	32.74 (19.46, 50.33)	52.62 (41.72, 64.12)	-19.7 (-33.1, -3.71)	0.01
Oromia	52.29 (10.05, 86.53)	43.06 (9.06, 80.55)	7.34 (-37.04, 49.91)	0.63
Sidama	73.18 (43.46, 88.83)	80.55 (49.84, 93.61)	-8.61 (-38.47, 28.53)	0.35
Snnp	51.72 (9.54, 85.39)	50.55 (13.4, 79.82)	1.43 (-47.36, 40.82)	0.53
Somali	28.48 (4.32, 66.83)	16.17 (2.49, 56.34)	9.8 (-34.59, 51.61)	0.69
Tigray	66.62 (31.49, 88.55)	62.73 (34.59, 84.63)	3.44 (-30.45, 35.6)	0.58

Kenya had the biggest absolute national change in the estimated percentage change of mDFPS coverage between the two periods. It also had a high probability that the absolute difference was greater than zero. The EP of the change of mDFPS being greater than zero was closer to 0.60 for Ethiopia and Nigeria.

At the sub-national level, in Ethiopia, only one region, Gambela, had a high EP probability (0.90) that the change in mDFPS coverage proportion improved in the second period of 2019 compared to 2016 (Table 5). In Kenya, the counties that had an EP of 0.9 or more probability of improving in mDFPS were 22 counties (Baringo, Bomet, Bungoma, Elgeyo-Marakwet, Homa Bay, Isiolo, Kajiado, Kericho, Kilifi, Kisumu, Laikipia, Mandera, Migori, Nakuru, Narok, Nyandarua, Taita Taveta, Trans Nzoia, Turkana, Vihiga, Wajir, West Pokot) between the year 2014 compared to 2022 (Table S1). While in Nigeria, the states with an EP of 0.9 or more probability of improving in mDFPS were 9 (Adamawa, Buachi, Gombe, Jigawa, Kano, Katsina, Kebbi, Sokoto and Zamfara) between the years 2013 compared to 2018 (Table S2). Conversely, the areas that had a very low EP probability ( < = 0.1) of improvement in coverage of mDFPS were likely to have had a reversal in coverage of mDFPS in the later period (Table 5 and Supplementary material Tables S1 & S2).

## Discussion

The results from multilevel modelling varied among the three countries, except for a few determinants, emphasising the uniqueness of each country. For instance, in Ethiopia, women without an education or with primary-level education had similar odds of mDFPS as those with secondary or higher-level education. In Kenya and Nigeria, women without an education had lower odds of mDFPS. This suggests that education may not contribute to mDFPS uptake inequality in Ethiopia compared to Kenya and Nigeria. In all three countries, women from well-to-do households had higher odds of mDFPS, highlighting that household wealth remains an important determinant of inequality [1, 4]. Muslims consistently had lower odds of mDFPS than women from other denominations; this provides an opportunity for tailored interventions to improve this population’s access and use of modern contraception [6].

In all three countries, geographic variations in the proportion of mDFPS were observed. The pattern of geographic variation was similar for the first and second periods, with a higher proportion of mDFPS seen in the relatively affluent sub-national areas. The analysis results investigating whether there was an improvement in the proportion of mDFPS, show that both Ethiopia and Nigeria had modest improvements. In contrast, Kenya increased its coverage of mDFPS between the two periods; 22 out of the 47 states improved coverage of mDFPS over the two periods, 2014 to 2022, with 9 counties likely to have exceeded the minimum WHO recommendation threshold in the second period, 2022. In the sub-national analysis in Ethiopia, Gambela region (out of 12 regions) was identified as the only region experienced a significant increase in its mDFPS coverage, over the two periods, 2016 to 2019. In Nigeria, 9 (out of 36 states) were identified as having had an increase in mDFPS coverage from 2013 to 2018, all from the northern region. In Ethiopia and Nigeria, most administrative areas had only slight improvements or stagnated in mDFPS coverage. It should be noted that even in regions where improvements were registered, the level of coverage of mDFPS was still below the WHO target. This can be seen on the exceedance maps of the WHO recommended 75% coverage that barely changed between the two periods.

The current analysis results indicate that demographic, geographic, and socioeconomic factors are important in determining who has greater coverage of mDFPS. These findings are consistent with previous studies that have analysed data on mDFPS [5, 44]. The geospatial analysis identified areas that still need to meet the WHO mDFPS coverage target. The geospatial analysis of changes in the coverage of mDFPS between the two time periods identified regions that have experienced either an increase or a decline and those that have been stagnant. These findings can guide targeted interventions that can address the unique needs of local communities instead of generalised interventions that local evidence does not drive [3, 20, 39, 53].

Administrative areas with declining or stagnant mDFPS coverage should be prioritised for interventions to improve the situation. In areas that have shown progress, efforts should be sustained to maintain and accelerate the gains made [45]. Even though we are advocating for interventions to increase the level of coverage of mDFPS, the rights of the individual and the couples and communities must be respected as enshrined in the 1994 International Conference on Population and Development (ICPD) conference [22]. It is important to acknowledge that some women may not start using modern contraceptive methods soon, even if they have an unmet need for modern contraception methods due to reasons such as perceived low risk of pregnancy or perceived cultural, social, or health concerns and preference for traditional methods [15, 42]. On the other hand, it’s worth noting that a portion of the unmet need for contraceptives may reflect a desire for modern contraception use, making these individuals more likely to adopt it when made available and accessible [15, 17].

Access and utilisation of modern contraceptive products can also be affected by conflicts and climatic emergencies [35, 46]. Women living in areas with ongoing conflict or climatic catastrophic events have reduced usage of modern contraception [35, 46]. In general, conflicts and climate change events can pose significant challenges to countries in meeting the demand for family planning services among women. When such events occur, the health system may be overwhelmed and unable to provide the necessary services that enable women to access and use modern contraception methods [46]. These challenges can have a particularly profound negative effect in regions and countries with already vulnerable healthcare infrastructure, like most countries in the SSA region [21, 48].

In Ethiopia, the Tigray region (and other regions such as Amhara, Afar and Oromia) has been at war since November 2020 until the time of writing this paper. Since the data analysed in this work was from a pre-conflict period, the situation is likely to have changed [21]. The Afar, Oromia, Somali and Harari regions are prone to drought and were also identified as having low mDFPS coverage in this analysis [7, 24, 28]. Similarly, in Kenya, most counties (Garisa, Isiolo, Mandera, Marsabit, Tana River, Samburu, Turkana and Wajir) with a history of drought were also identified as having a low coverage of mDFPS. When communities live in resource-constrained conditions, they prioritise survival and hence have low utilisation of health services [33, 49].

Furthermore, the northern Kenyan counties also face conflicts as communities are forced to fend for scarce resources. This results in displaced communities, further exacerbating the issues of health service utilisation [28]. To promote the use of modern contraception methods in such areas, it is necessary to take a holistic approach that addresses the challenges caused by conflict and climate change shocks [41].

In Nigeria, the northern states have been at war since 2009 [38]; our analysis shows that most of the northern states have low coverage of mDFPS. The region is also predominantly Muslim [43]. Our analysis and work from elsewhere show that Muslim women have lower odds of modern contraception use than women from Christian-based religions in the SSA [6]. In addition, misconceptions about modern contraception methods discourage women from using these methods in northern Nigeria. Some documented misconceptions include the belief that modern contraception can affect a woman’s ability to bear children and can cause cancer [26]. Work being done by humanitarian organisations can be alluded to as having helped to improve the level of mDFPS between the two time periods in the northern states, but there is more work that needs to be done to improve healthcare services in the region [27, 50]. Overall, Nigeria is one of the countries in SSA with a low modern contraception prevalence rate. In 2017, the Healthy Policy Plus initiative identified insufficient domestic funding as one of the challenges affecting Nigeria’s family planning programmes [23].

### Study strengths and limitations

The study had several strengths. Firstly, we employed a multilevel modelling approach to identify the disparities in mDFPS coverage driven by demographic, geographic, and socio-economic factors. This modelling process helped us identify the cluster and regional-level residual variance, indicating the presence of spatial variation. To account for this, we performed a spatial analysis using geostatistical models. This enabled us to calculate the adjusted proportion of mDFPS for each administrative area and determine the exceedance probability of each area, highlighting areas which exceeded the WHO mDFPS coverage minimum target of 75%. Additionally, our analysis of two time periods, which corresponded to a before and after period, allowed us to assess the progress made and identify areas that have stalled or retrogressed in their mDFPS coverage. The methods used in this study can be replicated for other SDG indicators and other important indicators to identify underserved communities, assisting policymakers and governments in directing their efforts more effectively.

Our research also had certain limitations. Despite our efforts, we could not find nationally representative data from 2015, when the UN established the SDG goals. Instead, we used the data that was closest to 2015. Similarly, for the latest year, we relied on the most recent data available for these countries. Thus, the time between the assessment years was different among the included countries, affecting the comparability of the results between the three countries. A complete spatial-temporal approach was impossible due to limited available data, which only spanned two time points. It is hard from this analysis to tell the actual reasons why the proportion of mDFPS reduced or improved in the areas that it did; hence, further studies are required in these areas. The survey responses may have been affected by social desirability bias, in which respondents provided answers that they believed were more favourable. It is also possible that the survey participants might have had recall bias; where they could have not accurately remembered the information they were being asked. Also, methodologies used between the two survey periods could have had changed, making direct comparisons challenging. Caution is necessary when generalizing these results to other countries, as their contexts may differ from the countries included in this analysis. The predicted posterior 95% CI’s were wide, but the uncertainty is comparable to DHS spatial analysis guidance recommendation [13]. Despite these limitations, our analysis provides valuable evidence and fills an essential gap in the available literature.

## Conclusion

Our analysis utilised multilevel modelling, geospatial analysis and comparison of data from three countries over two time periods. The results showed the presence of demographic, geographic and socio-economic disparities in the mDFPS, and that some areas have made progress while others have retrogressed but the majority have remained stagnant. The majority of sub-national level areas have mDFPS that are below the WHO recommendation of 75%. Our findings will be important in providing valuable insights for policymakers and governments on how they can effectively target their interventions to improve uptake of modern contraception among women who have a demand for it. By providing much-needed evidence, this analysis contributes to countries’ efforts to assess their progress in meeting the SDG indicator 3.7.1.

### Supplementary Information

Supplementary Material 1.

## Data Availability

The datasets analysed for this study are publicly available at https://dhsprogram.com/data/.

## Abbreviations

CI: Credible interval
CCI: Crude confidence interval
DHS: Demographic and Health Survey
EP: Exceedance probability
ICPD: International Conference on Population and Development
mDFPS: Demand for family planning satisfied with modern methods
MDG: Millennium Development Goals
MPC: Mean posterior change
OR: Odds ratio
PMA: Performance Monitoring for Action
SDG: Sustainable Development Goal
SRH: Sexual Reproductive Health
SSA: Sub-Sahara Africa
UN: United Nations
WHO: World Health Organisation
