# Supplementary material for: Unravelling factors influencing demand for modern contraception and evaluating coverage progress since 2015 in Ethiopia, Kenya, and Nigeria: insights from multilevel and geostatistical modelling
Source: BMC Public Health. 2024 Jul 11;24:1855. doi: 10.1186/s12889-024-19387-9 (PMC11238496; doi:10.1186/s12889-024-19387-9)
Supplement: Supplementary file 1 — Supplementary Material 1. [file 12889_2024_19387_MOESM1_ESM.docx]

**Supplementary material**

**Table S1: Table showing Kenya’s county-level proportion of mDFPS (2014-2022) with 95% credible intervals (CI), mean posterior change (MPC), percentage change, and exceedance probability of increase of greater than zero of mDFPS from 2014 to 2022. Estimates based on adjusted Bayesian geostatistical models.**

| **County** | **% mDFPS 2022**  **(95% CI)** | **% mDFPS 2014**  **(95% CI)** | **Mean posterior change (MPC) in mDFPS**  **(95% CI)** | **Probability of increase of mDFPS from 2014 to 2022** |
| --- | --- | --- | --- | --- |
| Baringo | 63.42 (40.1, 76.14) | 44.49 (17.32, 64.06) | 18.24 (1.48, 38.16) | 0.98 |
| Bomet | 71.77 (60.56, 79.45) | 63.05 (40.1, 72.72) | 9.12 (-2.82, 28.79) | 0.93 |
| Bungoma | 75.6 (57.64, 82.26) | 70.4 (39.05, 77.77) | 6.1 (-4.29, 27.12) | 0.87 |
| Busia | 70.52 (53.07, 80.37) | 68.09 (58.07, 75.39) | 2.14 (-14.77, 14.32) | 0.62 |
| Elgeyo-Marakwet | 70.56 (52.7, 81.03) | 55.45 (29, 71.24) | 15.71 (0.39, 33.19) | 0.98 |
| Embu | 81.05 (71.6, 87.84) | 81.9 (72.07, 88.38) | -0.92 (-10.1, 9.22) | 0.41 |
| Garissa | 32.32 (12.26, 59.39) | 20.67 (5.92, 52.54) | 9.9 (-16.01, 36.55) | 0.79 |
| Homa Bay | 74.47 (59.91, 82.35) | 62.59 (44.9, 72.19) | 11.82 (-1.17, 27.45) | 0.96 |
| Isiolo | 33.89 (11.62, 72.11) | 19.69 (7.05, 55.38) | 13.22 (-8.55, 40.05) | 0.88 |
| Kajiado | 65.2 (35.65, 81.4) | 47.39 (19.35, 72.41) | 15.8 (-5.09, 39.37) | 0.93 |
| Kakamega | 75.29 (67.55, 81.55) | 71.97 (63.53, 78.55) | 3.31 (-5.48, 12.47) | 0.78 |
| Kericho | 74.45 (64.91, 81.31) | 66.75 (51.79, 75.2) | 7.74 (-2.76, 21.65) | 0.92 |
| Kiambu | 78.51 (70.59, 84.71) | 80.42 (68.76, 86.24) | -1.79 (-10.65, 10.33) | 0.36 |
| Kilifi | 58.76 (33.61, 75.56) | 37.47 (18.46, 60.89) | 19.03 (-2.52, 42.69) | 0.96 |
| Kirinyaga | 81.51 (68, 86.92) | 83.85 (64.14, 88.66) | -1.85 (-9.25, 11.4) | 0.32 |
| Kisii | 75.65 (68.38, 81.04) | 74.41 (66.81, 80.93) | 1.07 (-7.09, 9.57) | 0.60 |
| Kisumu | 73.88 (62.6, 80.98) | 67.21 (49.86, 75.05) | 6.92 (-3.52, 20.54) | 0.91 |
| Kitui | 69.13 (42.15, 83.37) | 60.76 (29.75, 82.63) | 5.92 (-14.2, 36.21) | 0.70 |
| Kwale | 59.91 (38.73, 75.96) | 51.87 (32.98, 66.18) | 7.89 (-13.92, 29.84) | 0.77 |
| Laikipia | 72.64 (51.07, 84.64) | 58.33 (35.87, 78.19) | 12.97 (-4.62, 31.77) | 0.92 |
| Lamu | 59.19 (36.93, 78.34) | 51.82 (28.21, 70.4) | 5.98 (-16.18, 37.91) | 0.70 |
| Machakos | 78.97 (68.71, 86.49) | 79.6 (65.07, 86.09) | -0.1 (-10.13, 10.69) | 0.49 |
| Makueni | 74.94 (60.69, 83.98) | 66.99 (42.74, 81.3) | 7.5 (-8.03, 30.36) | 0.80 |
| Mandera | 13.72 (4.89, 30.86) | 2.96 (0.5, 10.78) | 10.29 (-0.4, 27.36) | 0.97 |
| Marsabit | 19.9 (7.34, 49.26) | 21.51 (6.31, 50.43) | -1.23 (-31.64, 29.5) | 0.46 |
| Meru | 76.57 (57.31, 85.62) | 72.63 (42.9, 84.48) | 4.03 (-9.05, 27.8) | 0.70 |
| Migori | 73.91 (61.07, 82.08) | 58.18 (39.45, 71.53) | 15.07 (2.42, 32.33) | 0.99 |
| Mombasa | 68.27 (59.81, 75.87) | 70.39 (60.94, 77.19) | -1.87 (-11.91, 8.31) | 0.36 |
| Murang'a | 79.82 (70.33, 85.69) | 79.28 (62.64, 84.68) | 0.92 (-7.8, 13.8) | 0.58 |
| Nairobi | 77.31 (70.74, 82.4) | 82.28 (73.97, 86.71) | -4.83 (-11.91, 3.69) | 0.11 |
| Nakuru | 76.7 (65.97, 84.54) | 68.33 (54.41, 79.6) | 8.06 (-4.42, 22.55) | 0.90 |
| Nandi | 75.32 (66.14, 81.7) | 71.8 (61.67, 78.76) | 3.55 (-6.09, 13.1) | 0.77 |
| Narok | 61.14 (33.67, 78.08) | 43.69 (19.18, 64.47) | 16.72 (-3.64, 34.97) | 0.95 |
| Nyamira | 74.59 (67.23, 80.17) | 73.76 (65.42, 79.98) | 0.83 (-7.2, 9.12) | 0.58 |
| Nyandarua | 80 (71.34, 86.62) | 73.24 (61.65, 80.87) | 6.84 (-3.57, 18.34) | 0.90 |
| Nyeri | 81.18 (68.6, 87.46) | 78.56 (61.2, 85.84) | 2.57 (-6.97, 16.99) | 0.70 |
| Samburu | 31.2 (13.82, 59.21) | 31.32 (12.34, 51.43) | 2.12 (-26, 26.87) | 0.56 |
| Siaya | 66.77 (52.51, 76.1) | 65.81 (49.64, 73.85) | 1.02 (-14.13, 18.34) | 0.56 |
| Taita Taveta | 73.32 (50.68, 86.06) | 57.54 (32.21, 76.11) | 14.9 (-7.64, 39.46) | 0.91 |
| Tana River | 43.88 (23.17, 68.67) | 32.28 (15.04, 54.59) | 10.81 (-14.82, 40.75) | 0.78 |
| Tharaka-Nithi | 78.94 (66.25, 86.37) | 76.8 (56.99, 86.25) | 1.88 (-8.94, 20.17) | 0.62 |
| Trans Nzoia | 75.95 (62.79, 83.57) | 61.44 (44.62, 72.54) | 14.23 (2.87, 27.94) | 0.99 |
| Turkana | 41.81 (20.35, 64.6) | 15.07 (5.18, 35.63) | 25.75 (-0.15, 49.68) | 0.97 |
| Uasin Gishu | 76.54 (66.83, 83.14) | 71.07 (60.42, 79.46) | 5.02 (-5.73, 18.04) | 0.81 |
| Vihiga | 74.26 (66.64, 81.07) | 68.73 (61.11, 75.41) | 5.58 (-2.56, 13.69) | 0.91 |
| Wajir | 20.06 (7.36, 40.85) | 7.96 (1.9, 21.38) | 11.47 (-4, 32.14) | 0.93 |
| West Pokot | 39.26 (16.92, 70.48) | 19.48 (5.46, 51.12) | 18.37 (0.41, 36.03) | 0.98 |

**Table S2: Table showing Nigeria's state-level proportion of mDFPS (2013-2018) with 95% credible intervals (CI), mean posterior change (MPC), percentage change, and probability exceedance probability of increase of greater than zero of mDFPS from 2013 to 2018. Estimates based on adjusted Bayesian geostatistical models.**

| **State** | **% mDFPS 2018**  **(95% CI)** | **% mDFPS 2013**  **(95% CI)** | **Mean posterior change (MPC) in mDFPS**  **(95% CI)** | **Probability of increase of mDFPS from 2013 to 2018** |
| --- | --- | --- | --- | --- |
| Abia | 28.74 (21, 39.35) | 41.44 (30.84, 51.8) | -12.59 (-24.68, 1.39) | 0.04 |
| Adamawa | 40.4 (13.76, 64.57) | 9.73 (2.86, 22.35) | 30.57 (4.04, 52.01) | 0.99 |
| Akwa Ibom | 32.44 (23.11, 42.85) | 39.59 (28.08, 50.15) | -7.39 (-20.42, 9.87) | 0.17 |
| Anambra | 32.16 (20.36, 41.49) | 35.44 (23.22, 45.93) | -3.51 (-14.96, 9.12) | 0.27 |
| Bauchi | 18.97 (6.97, 40.06) | 7.94 (2.17, 29.99) | 8.91 (-6.63, 30.27) | 0.89 |
| Bayelsa | 12.21 (3.97, 30.63) | 37.08 (26.99, 49.42) | -24.46 (-39.31, -3.28) | 0.01 |
| Benue | 36.03 (21.31, 52.38) | 31.76 (17.71, 49.79) | 4.52 (-16.93, 22.47) | 0.67 |
| Borno | 8.66 (0.47, 40.13) | 7.6 (0.78, 25.51) | 0.7 (-13.63, 28.06) | 0.56 |
| Cross River | 32.57 (16.87, 53.69) | 33.8 (19.16, 51.34) | 0.16 (-25.56, 19.97) | 0.51 |
| Delta | 24.4 (8.03, 39.15) | 43.29 (29.28, 58.34) | -19.94 (-37.31, -2.35) | 0.01 |
| Ebonyi | 24.89 (17.13, 35.36) | 27.31 (16.97, 43.21) | -2.81 (-17.21, 12.08) | 0.35 |
| Edo | 26.43 (14.68, 38.84) | 43.21 (28.12, 57.26) | -16.64 (-30.57, -2.02) | 0.01 |
| Ekiti | 47.49 (34.54, 57.1) | 52.58 (40.51, 65.45) | -5.83 (-18.84, 7.25) | 0.19 |
| Enugu | 39.43 (24.6, 54.53) | 39.86 (25.03, 55.1) | -0.31 (-14.07, 14.26) | 0.48 |
| FCT - Abuja | 35.9 (15.53, 62.94) | 35.51 (14.05, 63.85) | 0.12 (-13.48, 15.43) | 0.51 |
| Gombe | 41.34 (23.22, 61.35) | 9.46 (2.55, 31.29) | 30.18 (12.68, 46.27) | 1.00 |
| Imo | 28.92 (21.02, 36.9) | 36.06 (23.86, 48.72) | -7.22 (-22.11, 7.82) | 0.19 |
| Jigawa | 14.85 (6.79, 31.5) | 3.45 (1.19, 10.23) | 11.09 (2.05, 26.57) | 0.99 |
| Kaduna | 44.89 (17.65, 69.65) | 54.6 (10.59, 79.75) | -6.3 (-30.44, 22.53) | 0.32 |
| Kano | 18.83 (8.7, 38.37) | 5.76 (1.51, 27.08) | 12.21 (-5.47, 29.5) | 0.95 |
| Katsina | 15.97 (7.49, 32.96) | 6.66 (1.96, 25.35) | 8.41 (-4.31, 23.55) | 0.92 |
| Kebbi | 13.66 (4.12, 37.81) | 3.63 (0.82, 16.65) | 9.03 (-3.87, 32.43) | 0.93 |
| Kogi | 31.94 (16.07, 50.29) | 33.61 (16.52, 53.16) | -1.66 (-21.87, 18.71) | 0.44 |
| Kwara | 24.81 (9.01, 46.19) | 38.19 (11.83, 65.49) | -14.24 (-36.02, 15.73) | 0.16 |
| Lagos | 38.37 (21.06, 49.46) | 47.64 (22.45, 59.02) | -8.4 (-23.05, 7.83) | 0.12 |
| Nassarawa | 29.28 (15.12, 51.55) | 34 (15.94, 56.98) | -4.74 (-23.91, 17.62) | 0.33 |
| Niger | 18.82 (6.49, 42.05) | 14.71 (2.9, 42.18) | 3.24 (-16.82, 25.12) | 0.64 |
| Ogun | 32.67 (15.4, 47.49) | 36.45 (12.66, 54.19) | -3.38 (-20.9, 15.97) | 0.35 |
| Ondo | 30.99 (13.5, 50.16) | 41.65 (27.98, 55.35) | -10.33 (-26.55, 6.47) | 0.11 |
| Osun | 46.8 (32.57, 59.18) | 53.54 (37.36, 67.35) | -7.03 (-23.2, 10.46) | 0.22 |
| Oyo | 25.89 (12.03, 46.17) | 40.77 (21.39, 57.86) | -14 (-33.12, 9.27) | 0.10 |
| Plateau | 38.07 (14.25, 66.51) | 35.45 (16.78, 64.91) | 2.36 (-18.82, 21.65) | 0.59 |
| Rivers | 38.03 (22.55, 51.82) | 37.77 (27.92, 49.33) | 0.59 (-19.2, 14.92) | 0.53 |
| Sokoto | 6.86 (1.74, 29.15) | 2.92 (0.53, 15.45) | 3.48 (-4.1, 20.6) | 0.86 |
| Taraba | 20.34 (6.8, 49.95) | 18.6 (5.03, 38.34) | 2.27 (-17.82, 31.51) | 0.59 |
| Yobe | 4.41 (0.6, 23.55) | 2.7 (0.52, 9.45) | 1.64 (-4.22, 18.79) | 0.73 |
| Zamfara | 11.45 (2.56, 33.58) | 5.5 (1.56, 17.38) | 5.52 (-3.88, 22.71) | 0.86 |

**Table S3: Adjusted odds ratios (aOR) for demand for family planning satisfied with modern contraception methods in Ethiopia (DHS), Kenya (DHS) and Nigeria (DHS) based on the using Bayesian geostatistical logistic regression (Equation 2). Based on the first survey.**

| **Characteristic** | **Ethiopia (year: 2016)** | **Kenya (year: 2014)** | **Nigeria (year: 2013)** |
| --- | --- | --- | --- |
|  | **aOR (95% CI)** | **aOR (95% CI)** | **aOR (95% CI)** |
| **Women education years** | 2.25 (1.32, 3.83) | 3.43 (2.10, 6.47) | 10.83 (6.12,19.83) |
| **Population density** | 1.15 (1.07, 1.24) | 1. 05 (0.99, 1.12) | 1.09 (1.03, 1.15) |
| **Mean parity** | 0.68 (0.64, 0.72) | 0.84 (0.81, 0.88) | 0.89 (0.86, 0.92) |
| **Probability seeking care at a health facility** | 1.06 (0.59, 1.88) | 0.63 (0.28, 1.56) | 1.58 (1.18, 2.11) |
| **Percentage of people living below a one USA dollar^1^** |  | 0.27 (0.12, 0.80) | 2.11 (0.85, 5.23) |

^1^Percentage of people living below US $1-dollar variable was not available for Ethiopia

**
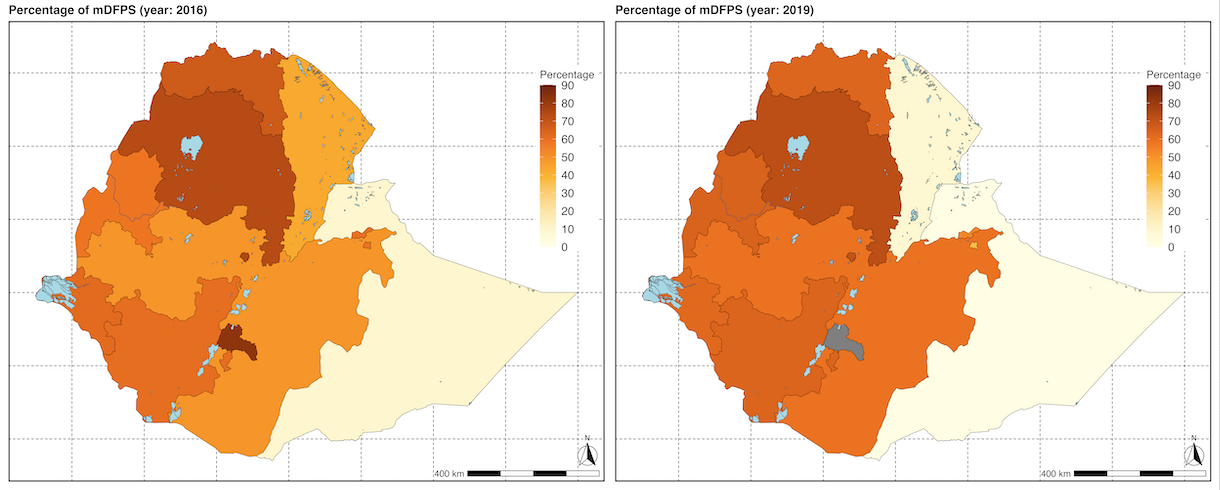
**

**Figure S1: Percentage of demand for family planning satisfied with modern contraception methods (mDFPS) for Ethiopia. Estimates based on unadjusted weighted averages. *Top left panel:*** Map of the percentage of demand for family planning satisfied with modern contraception methods (mDFPS) in Ethiopia in 2016 (DHS).  ​***Top right panel:*** Map of the crude percentage of mDFPS in Ethiopia in 2019. Note: data was not collected in Sidama in 2019 (PMA).

**
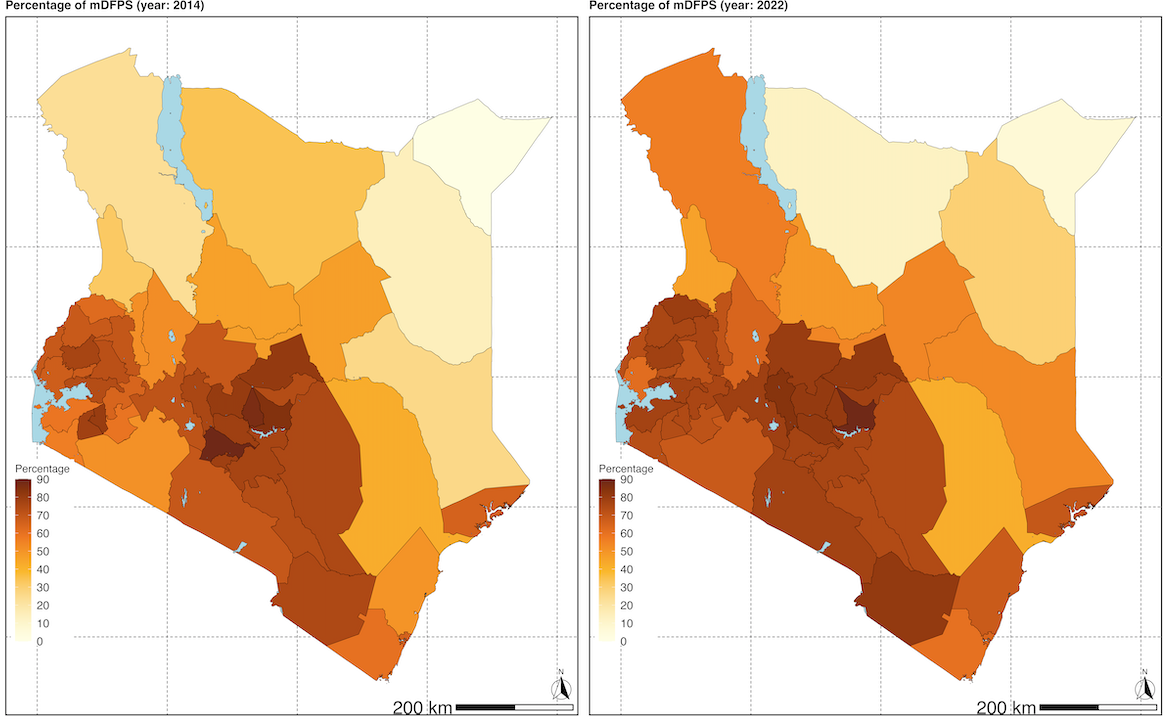
**

**Figure S2: Percentage of demand for family planning satisfied with modern contraception methods (mDFPS) for Kenya. Estimates based on unadjusted weighted averages. *Top left panel:*** Map of the percentage of demand for family planning satisfied with modern contraception methods (mDFPS) in Kenya in 2014 (DHS).  ​***Top right panel:*** Map of the crude percentage of mDFPS in Kenya in 2022 (DHS).

**
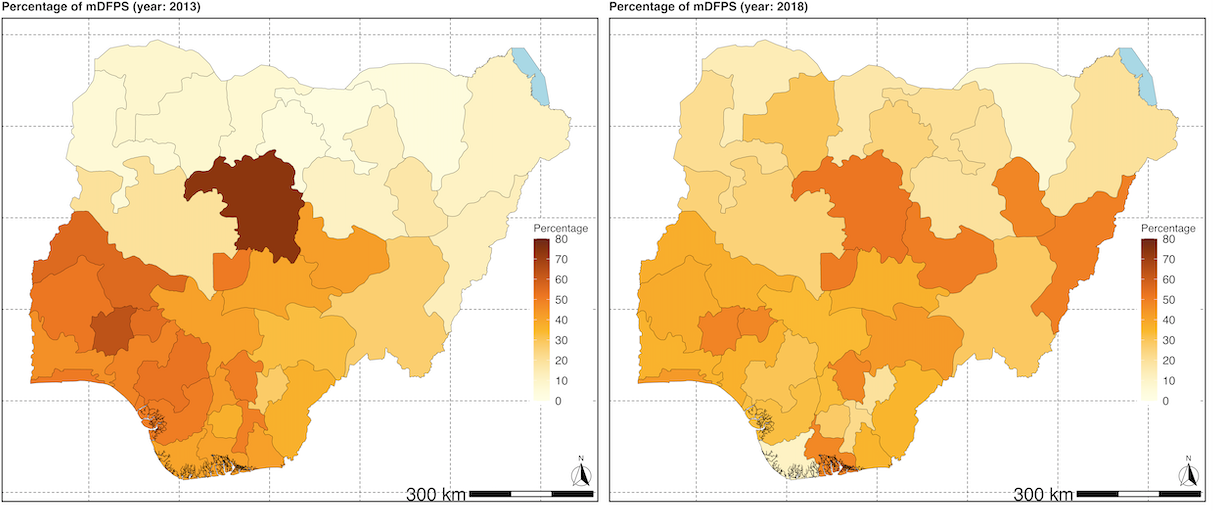
**

**Figure S3: Percentage of demand for family planning satisfied with modern contraception methods (mDFPS) for Nigeria. Estimates based on unadjusted weighted averages. *Top left panel:*** Map of the percentage of demand for family planning satisfied with modern contraception methods (mDFPS) in Nigeria in 2013 (DHS).  ​***Top right panel:*** Map of the crude percentage of mDFPS in Nigeria in 2018 (DHS).

**Table S4: Coverage of demand for family planning satisfied with modern methods (mDFPS) by characteristics of respondents based on latest standard DHS data for Ethiopia, 2016. Both counts and row-wise percentages were weighted to adjust for survey design.**

| **Characteristic** | **Overall, N = 6,355^1^** | **No, N = 2,456^1^** | **Yes, N = 3,899^1^** | p-value^2^ |
| --- | --- | --- | --- | --- |
| **Residence** |  |  |  | <0.001 |
| Urban | 1,208 (100%) | 264 (21.83%) | 944 (78.17%) |  |
| Rural | 5,148 (100%) | 2,193 (42.60%) | 2,955 (57.40%) |  |
| **Age groups** |  |  |  | <0.001 |
| 15-24 | 1,482 (100%) | 511 (34.49%) | 971 (65.51%) |  |
| 25-34 | 2,917 (100%) | 1,074 (36.82%) | 1,843 (63.18%) |  |
| 35-49 | 1,957 (100%) | 871 (44.53%) | 1,086 (55.47%) |  |
| **Level of education** |  |  |  | <0.001 |
| Secondary or higher | 799 (100%) | 187 (23.36%) | 612 (76.64%) |  |
| Primary | 1,931 (100%) | 693 (35.87%) | 1,238 (64.13%) |  |
| No education | 3,626 (100%) | 1,577 (43.50%) | 2,049 (56.50%) |  |
| **Sex of household head** |  |  |  | 0.4 |
| Male | 5,357 (100%) | 2,052 (38.31%) | 3,305 (61.69%) |  |
| Female | 999 (100%) | 404 (40.47%) | 595 (59.53%) |  |
| **Wealth index** |  |  |  | <0.001 |
| Poorest | 970 (100%) | 549 (56.59%) | 421 (43.41%) |  |
| Poorer | 1,263 (100%) | 589 (46.63%) | 674 (53.37%) |  |
| Middle | 1,326 (100%) | 522 (39.33%) | 805 (60.67%) |  |
| Richer | 1,294 (100%) | 423 (32.68%) | 871 (67.32%) |  |
| Richest | 1,503 (100%) | 374 (24.91%) | 1,129 (75.09%) |  |
| **Marital status** |  |  |  | <0.001 |
| In union | 5,949 (100%) | 2,345 (39.42%) | 3,604 (60.58%) |  |
| Never in union | 138 (100%) | 51 (36.86%) | 87 (63.14%) |  |
| Formerly in union | 269 (100%) | 61 (22.54%) | 208 (77.46%) |  |
| **Religion group** |  |  |  | <0.001 |
| Islam | 1,883 (100%) | 1,080 (57.36%) | 803 (42.64%) |  |
| Orthodox | 2,896 (100%) | 848 (29.27%) | 2,049 (70.73%) |  |
| Other | 79 (100%) | 57 (72.93%) | 21 (27.07%) |  |
| Other christians | 1,497 (100%) | 471 (31.46%) | 1,026 (68.54%) |  |
| **Parity group** |  |  |  | <0.001 |
| None | 552 (100%) | 174 (31.53%) | 378 (68.47%) |  |
| 1-2 | 1,807 (100%) | 497 (27.53%) | 1,309 (72.47%) |  |
| 3-4 | 1,598 (100%) | 543 (33.97%) | 1,055 (66.03%) |  |
| 5+ | 2,399 (100%) | 1,242 (51.77%) | 1,157 (48.23%) |  |
| ^1^n (%) | | | | |
| ^2^chi-squared test with Rao & Scott's second-order correction | | | | |

**Table S5: Coverage of demand for family planning satisfied with modern methods (mDFPS) by characteristics of respondents based on latest standard DHS data for Kenya, 2022. Both counts and row-wise percentages were weighted to adjust for survey design.**

| Characteristic | Overall, N = 9,490^1^ | No, N = 2,412^1^ | Yes, N = 7,078^1^ | p-value^2^ |
| --- | --- | --- | --- | --- |
| **Residence** |  |  |  | 0.2 |
| Urban | 3,819 (100%) | 930 (24.34%) | 2,890 (75.66%) |  |
| Rural | 5,671 (100%) | 1,482 (26.13%) | 4,189 (73.87%) |  |
| **Age groups** |  |  |  | <0.001 |
| 15-24 | 2,289 (100%) | 733 (32.01%) | 1,556 (67.99%) |  |
| 25-34 | 3,777 (100%) | 829 (21.96%) | 2,947 (78.04%) |  |
| 35-49 | 3,424 (100%) | 849 (24.81%) | 2,575 (75.19%) |  |
| **Level of education** |  |  |  | <0.001 |
| Secondary or higher | 5,261 (100%) | 1,326 (25.21%) | 3,935 (74.79%) |  |
| Primary | 3,859 (100%) | 884 (22.91%) | 2,975 (77.09%) |  |
| No education | 370 (100%) | 201 (54.36%) | 169 (45.64%) |  |
| **Sex of household head** |  |  |  | 0.002 |
| Male | 6,296 (100%) | 1,513 (24.02%) | 4,784 (75.98%) |  |
| Female | 3,194 (100%) | 899 (28.15%) | 2,295 (71.85%) |  |
| **Wealth index** |  |  |  | <0.001 |
| Poorest | 1,344 (100%) | 463 (34.45%) | 881 (65.55%) |  |
| Poorer | 1,725 (100%) | 403 (23.36%) | 1,322 (76.64%) |  |
| Middle | 1,797 (100%) | 427 (23.78%) | 1,370 (76.22%) |  |
| Richer | 2,188 (100%) | 550 (25.13%) | 1,638 (74.87%) |  |
| Richest | 2,436 (100%) | 569 (23.34%) | 1,868 (76.66%) |  |
| **Marital status** |  |  |  | <0.001 |
| In union | 7,089 (100%) | 1,792 (25.28%) | 5,297 (74.72%) |  |
| Never in union | 1,394 (100%) | 427 (30.67%) | 966 (69.33%) |  |
| Formerly in union | 1,007 (100%) | 192 (19.04%) | 816 (80.96%) |  |
| **Religion group** |  |  |  | <0.001 |
| Muslim | 363 (100%) | 163 (45.04%) | 199 (54.96%) |  |
| Catholic | 1,863 (100%) | 485 (26.03%) | 1,378 (73.97%) |  |
| Other | 372 (100%) | 123 (32.94%) | 250 (67.06%) |  |
| Other christians | 6,891 (100%) | 1,641 (23.81%) | 5,251 (76.19%) |  |
| **Parity groups** |  |  |  | <0.001 |
| None | 948 (100%) | 416 (43.86%) | 532 (56.14%) |  |
| 1-2 | 3,837 (100%) | 834 (21.72%) | 3,003 (78.28%) |  |
| 3-4 | 2,999 (100%) | 643 (21.43%) | 2,356 (78.57%) |  |
| 5+ | 1,706 (100%) | 520 (30.46%) | 1,186 (69.54%) |  |
| ^1^n (%) | | | | |
| ^2^chi-squared test with Rao & Scott's second-order correction | | | | |

**Table S6: Coverage of demand for family planning satisfied with modern methods (mDFPS) by characteristics of respondents based on latest standard DHS data for Nigeria, 2018. Both counts and row-wise percentages were weighted to adjust for survey design.**

| **Characteristic** | **Overall, N = 12,331^1^** | **No, N = 7,934^1^** | **Yes, N = 4,397^1^** | **p-value^2^** |
| --- | --- | --- | --- | --- |
| **Residence** |  |  |  | <0.001 |
| Urban | 6,625 (100%) | 3,945 (59.54%) | 2,680 (40.46%) |  |
| Rural | 5,705 (100%) | 3,989 (69.92%) | 1,716 (30.08%) |  |
| **Age groups** |  |  |  | 0.004 |
| 15-24 | 2,517 (100%) | 1,697 (67.43%) | 820 (32.57%) |  |
| 25-34 | 4,880 (100%) | 3,051 (62.53%) | 1,829 (37.47%) |  |
| 35-49 | 4,933 (100%) | 3,185 (64.56%) | 1,748 (35.44%) |  |
| **Level of education** |  |  |  | <0.001 |
| Secondary or higher | 7,339 (100%) | 4,256 (58.00%) | 3,082 (42.00%) |  |
| Primary | 2,053 (100%) | 1,323 (64.46%) | 730 (35.54%) |  |
| No education | 2,939 (100%) | 2,354 (80.10%) | 585 (19.90%) |  |
| **Sex of household head** |  |  |  | 0.11 |
| male | 10,347 (100%) | 6,699 (64.75%) | 3,648 (35.25%) |  |
| female | 1,984 (100%) | 1,235 (62.24%) | 749 (37.76%) |  |
| **Wealth index** |  |  |  | <0.001 |
| Poorest | 1,339 (100%) | 1,088 (81.30%) | 250 (18.70%) |  |
| Poorer | 1,771 (100%) | 1,297 (73.23%) | 474 (26.77%) |  |
| Middle | 2,395 (100%) | 1,592 (66.47%) | 803 (33.53%) |  |
| Richer | 3,253 (100%) | 1,961 (60.28%) | 1,292 (39.72%) |  |
| Richest | 3,573 (100%) | 1,996 (55.86%) | 1,577 (44.14%) |  |
| **Marital status** |  |  |  | <0.001 |
| In union | 10,337 (100%) | 6,834 (66.12%) | 3,503 (33.88%) |  |
| Never in union | 1,624 (100%) | 918 (56.49%) | 707 (43.51%) |  |
| Formerly in union | 369 (100%) | 182 (49.23%) | 187 (50.77%) |  |
| **Religion group** |  |  |  | <0.001 |
| Islam | 5,077 (100%) | 3,624 (71.38%) | 1,453 (28.62%) |  |
| Catholic | 1,458 (100%) | 906 (62.17%) | 552 (37.83%) |  |
| Other | 45 (100%) | 38 (84.03%) | 7 (15.97%) |  |
| Other christian | 5,750 (100%) | 3,365 (58.53%) | 2,385 (41.47%) |  |
| **Parity group** |  |  |  | <0.001 |
| None | 1,440 (100%) | 832 (57.77%) | 608 (42.23%) |  |
| 1-2 | 2,991 (100%) | 1,914 (63.97%) | 1,078 (36.03%) |  |
| 3-4 | 3,301 (100%) | 1,998 (60.55%) | 1,302 (39.45%) |  |
| 5+ | 4,598 (100%) | 3,190 (69.37%) | 1,408 (30.63%) |  |
| ^1^n (%) | | | | |
| ^2^chi-squared test with Rao & Scott's second-order correction | | | | |
